# Long-term effects on immunological, inflammatory markers, and HIV-1 reservoir after switching to a two-drug versus maintaining a three-drug regimen based on integrase inhibitors

**DOI:** 10.3389/fimmu.2024.1423734

**Published:** 2024-07-11

**Authors:** Abraham Saborido-Alconchel, Ana Serna-Gallego, María Trujillo-Rodriguez, Esperanza Muñoz-Muela, Ana I. Álvarez-Ríos, Carmen Lozano, Silvia Llaves-Flores, Nuria Espinosa, Cristina Roca-Oporto, Marta Herrero, Cesar Sotomayor, Alicia Gutierrez-Valencia, Luis F. Lopez-Cortes

**Affiliations:** ^1^ Clinical Unit of Infectious Diseases, Microbiology and Parasitology, Institute of Biomedicine of Seville/Virgen del Rocio University Hospital/The Spanish National Research Council (CSIC)/University of Seville, Seville, Spain; ^2^ Department of Clinical Biochemistry, Institute of Biomedicine of Seville/Virgen del Rocio University Hospital/The Spanish National Research Council (CSIC)/University of Seville, Seville, Spain; ^3^ Primary Care Pharmacist Service, Sevilla Primary Care District, Seville, Spain

**Keywords:** HIV-1 infection, HIV-1 treatment, long-term, inflammation, antiretroviral therapy, dual therapy, triple therapy

## Abstract

**Objective:**

To compare the long-term effects on immune parameters, inflammation, and HIV-1 reservoir after switching to a two-drug (2DR) versus maintaining an integrase inhibitor (InSTI)-based three-drug regimen (3DR).

**Methods:**

Cross-sectional study in which HIV-1 treatment-naïve people started and maintained an InSTI-based 3DR or, at different times, switched to 2DR (dolutegravir or darunavir/cobicistat + lamivudine). CD4^+^ and CD8^+^ T-cell activation and exhaustion, plasma concentrations of hs-CRP, D-dimer, P-selectin, IL-1β, IL-6, TNF-α, IFN-γ, IP-10, sTNFR-I/II, MIP-1α/β, I-FABP, LBP, sCD14, sCD163, MCP-1, and cellular-associated HIV-1-DNA and -RNA were quantified by flow cytometry, different immunoassays, and droplet digital PCR, respectively. The U de Mann-Whitney test evaluated differences between 3DR and 2DR. Immune recovery was evaluated using a general linear model for repeated measures adjusted for different co-variables.

**Results:**

Fifty participants per group were included. The median time on 3DR was 82 months for the 3DR group and 30 months for the 2DR group, after which it switched to 2DR for a median of 57 months. We did not find differences between both groups in any of the parameters analyzed. Specifically, some values in 3DR and 2DR were hs-CRP, 0.92 mg/L (0.45–2.23) vs. 1.23 (0.61–2.38); D-dimer, 190.0 µg/L (150.0–370.0) vs. 190.0 (150.0–397.5); IL-6, 2.8 pg/mL (1.3–5.3) vs. 3.2 (2.1–4.7); sCD14, 4.5 ng/mL (3.3–6.2) vs. 5.0 (3.6–6.1), respectively, all p ≥ 0.399.

**Conclusion:**

In the long term, switching to 2DR does not negatively affect immunologic parameters, inflammatory markers, or HIV-1 reservoir.

**Clinical trial registration:**

identifier NCT04076423.

## 1 Introduction

Recent advances in ART have proven the non-inferiority of two-drug regimens (2DR) regarding virological efficacy and immune reconstitution compared to traditional three-drug regimens (3DR) (1, 2). On the other hand, lower drug concentrations in lymphoid tissues may result in incomplete suppression of viral replication that would contribute to chronic immune activation and inflammation (cIA/I) (3, 4). In this context, some studies have suggested that switching to 2DR is associated with a worse long-term inflammatory profile than maintaining a 3DR (5–8). Therefore, there is concern that switching to 2DR could not control tissular viral replication as well as 3DR, which would adversely impact the cIA/I and HIV-1 reservoir in the long term. Given its potential clinical implications, this needs to be clarified, as the increase in 2DR use, based on a reduction in drug exposure, the potential for lower toxicity, and the costs of a probably lifetime ART, could lead to long-term comorbidities compromising the quality of life and survival of PHIV (9). This study aims to compare the long-term effects on immune parameters, inflammatory markers, HIV-1 reservoir, and transcriptional activity of switching to a 2DR versus maintaining an integrase inhibitor (InSTI)-based 3DR.

## 2 Methods

### 2.1 Study design and outcomes

The Long-term TRIDUAL (Long-term TRIple versus DUAL therapy) was a cross-sectional, observational study carried out at Virgen del Rocío University Hospital in Spain. The primary endpoint was to evaluate long-term differences between 3DR and 2DR in T-cell activation and exhaustion, and plasma concentrations of soluble inflammatory markers. Additionally, we sought differences in the HIV reservoir measured as total cellular-associated HIV-1 DNA (HIV-1-DNA) and HIV-1-RNA, and immune recovery.

The study was conducted in accordance with the Declaration of Helsinki. The study was approved by the Ethics Committee for Clinical Research of the Virgen Macarena and Virgen del Rocío University Hospitals (CEI VM-VR_06/2021_N) and the Agencia Española del Medicamento y Productos Sanitarios. Spain. All participants provided signed informed consent. The protocol was registered at clinicaltrials.gov (NCT04076423).

### 2.2 Study population

Eligible participants were adult treatment-naïve PHIV who started ART with an InSTI-based plus two nucleos(t)ide reverse transcriptase inhibitors (NRTI) (3DR) after 01/01/2010, achieved an HIV-1 RNA <50 copies/mL during the first 24 weeks of treatment, and maintained it ≥95% of the determinations throughout the follow-up. Single blips were allowed, defined as a positive viral load <500 copies/mL preceded and followed by an undetectable viral load.

Exclusion criteria included pregnancy, active opportunistic infection, HBV or HCV coinfection, liver cirrhosis Child-Pugh stage A or higher, current or past treatment with drugs that could affect inflammatory markers or T-cell counts, and virological failure (VF), defined as two consecutive plasma HIV-1 RNA >200 copies/mL, or a single HIV-1 RNA level >200 copies/mL if followed by a loss of follow-up (10).

For the study purpose, participants classified as 3DR maintained an InSTI plus two NRTI until sampling. Treatment changes were allowed within each drug family due to the appearance of new drugs within the same family. By contrast, participants classified as 2DR started a 3DR with an InSTI plus two NRTI and, at different times switched to dolutegravir (DTG), 50 mg, or darunavir/cobicistat (DRV_c_), 800 mg/150 mg, plus lamivudine (3TC), 300 mg once daily, at the criteria of their attending physician, and maintained it at least four years until sampling.

Demographics and clinical data were obtained from standardized electronic medical records from all PHIV receiving care at our center, guaranteeing that the information handled does not contain personal data.

### 2.3 Procedures

CD4^+^ and CD8^+^ T-cell counts were determined with an FC 500 flow cytometer (Beckman Coulter). Plasma HIV-1 RNA levels were measured by quantitative PCR (Cobas AmpliPrep-Cobas TaqMan HIV-1 test, v 2.0 in the past and Cobas 6800 system from 2018 onward with a lower quantification limit of 20 copies/mL; Roche Diagnostics). Both determinations were routinely performed at each analytical control since the start of ART.

### 2.4 CD4^+^ and CD8^+^ T-cell immune activation and exhaustion and plasma inflammatory markers

CD4^+^ and CD8^+^ T-cell activation and exhaustion were assessed by the expression of CD38 and HLA-DR, and of PD1, TIGIT, and TIM-3 as detailed in **Supplementary Material**. For plasma soluble inflammatory makers, plasma samples were aliquoted and stored at -80°C until subsequent assays. β-2 microglobulin (β-2M), high sensitivity C-reactive protein (hs-CRP), D-dimer, interleukin (IL) -1β, IL-6, tumor necrosis factor-α (TNF-α), interferon-γ (IFN-γ), interferon gamma-induced protein 10 (IP-10), macrophage inflammatory protein (MIP)-1α/-1β, soluble tumor necrosis factor receptor (sTNFR) -I/-II, monocyte chemoattractant protein 1 (MCP-1), soluble CD14 (sCD14), soluble CD163 (sCD163), P-selectin, lipopolysaccharide-binding protein (LBP), and intestinal fatty-acid-binding proteins (I-FABP) were measured by different immunoassay kits, following the manufacturers’ instructions, as detailed in **Supplementary Material**.

### 2.5 HIV-1 reservoir and transcriptional activity

HIV-1-DNA and -RNA were quantified in purified CD4^+^ T cells by droplet digital PCR (ddPCR) (11) using the BIO-RAD QX200 Droplet Reader. CA-DNA and RNA were extracted from cryopreserved PBMC using the E.Z.N.A.™ DNA/RNA Kit (Omega, Bio-Tek). DNAse I (Invitrogen) was used to remove any DNA contamination from total RNA. The Qubit Assay kit (ThermoFisher Scientific) was used to measure concentrations of DNA and RNA carried at a concentration of 30 ng/µL. To reverse the RNA transcription and perform the ddPCR, the One-Step RT-ddPCR kit was used. For both CA HIV-1-DNA and RNA, we used a mixture of two primers in the viral 5′ long terminal repeat (5-LTR) and gag regions: Forward primer 1: `5-TGTGTGCCCGTCTGTTGTGT-3’ and reverse primer:`5-GCCGAGRCCTGCGTCGAGAG-3’ with a specific probe: `5-FAM-CAGTGGCGCCCGAACAGGGA-BHQ1-3’, and forward primer 2: `5-CATGTTTTCAGCATTATCAGAAGGA-3’ and reverse primer: `5-TGCTTGATGTCCCCCCACT-3’ with a specific probe: `5-VIC-CC ACCCCACAAGATTTAAACACCATGCTAA-BHQ1-3’ (12).

For the detection of 2LTR circles, the primers/probes used were: Forward primer: ‘5-AACTAGGGAACCCACTGCTTAAG-3’, reverse primer: ‘5-TCCACAGATCAAGGATATCTTGTC-3’ and probe: ‘5-FAM- ACACTACTTGAAGCACTCAAGGCAAGCTTT-BHQ1-3’. The RPP30 and TBP housekeeping genes were quantified to normalize the input DNA and RNA samples (13). Bio-Rad QuantaSoft software v. 1.7.4. was used to calculate the number of copies. The researchers involved in the laboratory techniques were blind to each participant’s treatment arm, so no subjective assessments were made.

### 2.6 Statistical analysis

For the sample size calculation, we used the estimated differences in the evolution of IL-6 (median, one pg/ml) and D-dimer (median 130 ng/ml) values between 3DR and 2DR after four years of switching in the study of Serrano-Villar et al. (data assumed from graphs) (8). Thus, for a power of 90% and an alpha error of 0.05, it would be necessary to include 49 participants per group in the case of IL-6 and 50 in the case of D-dimers [Ene 3.0 (e-Biometria, Madrid, Spain)].

Continuous measures are described by medians, interquartile ranges (IQR), and [ranges], and categorical variables with frequencies and percentages, respectively. The chi-square test was used to compare categorical variables. We used the U de Mann-Whitney test to detect the effects between 3DR and 2DR. A general linear model for repeated measures was used to compare the time courses of the CD4^+^ T-cell counts and CD4^+^/CD8^+^ ratios according to regimen strategy, adjusted for possible confounders, including age, sex, highest viral load before starting ART, first ART, and number of blips through the follow-up. The overall effect of each explanatory variable on the outcome variables, CD4^+^ T-cell count and CD4^+^/CD8^+^ ratio, was tested with the F-test and CI_95_. Statistical analyses were performed using the IBM software (SPSS v.26.0, Chicago, USA); p-values <0.05 were considered significant. Graphs were generated with GraphPad Prism Software, v.9.0.0.

## 3 Results

One hundred PHIV were enrolled between February 2022 and February 2023. All participants started ART with a 3DR based on an InSTI plus two NRTI. Fifty subjects maintained 3DR for a median of 82 months (75–91), all of them were on bictegravir, tenofovir alafenamide, and emtricitabine (BIC/TAF/FTC) when included and sampled (**Supplementary Figure 1**). A posteriori, one participant was excluded from the 3DR for not complying with the inclusion criteria. The total time on treatment of the remainder fifty participants (2DR group) was 88 months (77–94), of which a median of 30 months (20−38) was on an InSTI-based 3DR, and, afterward, switched to 2DR [DTG/3TC, 35 (70%) or DRV_c_ plus 3TC, 15 (30%)] during a median of 57 months (53–60; range, 48–65). The characteristics of the study participants are shown in **Table 1**.

**Table 1:** Characteristics of the study participants. Data are expressed as median (interquartile range) or n (%).

	3DR (n= 49)	2DR (n= 50)	p
**Male sex, n (%)**	48 (98.0)	47 (94.0)	0.617
**Age, years**	43 (37 − 53)	43 (38 – 49)	0.704
Risk for HIV infection, n (%)			
**MSM/Others**	44 (89.8) / 5 (10.0)	42 (84.0) / 8 (16.0)	0.330
Previous to first ART			
**CDC stage C**	5 (10.2)	2 (4.0)	0.579
**Plasma HIV-RNA, copies/mL**	71250 (26500–169000)	33100 (13250–73900)	0.019
**Plasma HIV-RNA >100000 copies/mL**	18 (36.7)	12 (24.0)	0.130
**CD4^+^ T cell count/μL**	407 (202–526)	407 (292–502)	0.663
**CD4^+^/CD8^+^ ratio**	0.44 (0.24–0.62)	0.41 (0.19–0.62)	0.779
First ART (3DR)			
EVGc/TAF/FTC (%)	37 (75.5)	37 (74.0)	0.793
DTG/ABV/3TC (%)	8 (16.3)	12 (24.0)	
DTG/TDF+FTC (%)	4 (8.1)	1 (2.0)	
Maintained 3DR			
BIC/TAF/FTC, (%)	49 (100)	**−**	**−**
Switch to 2DR			
DTG / DRVc + 3TC, n (%)	**−**	35 (70.0) / 15 (30.0)	**−**
At the time of sampling			
**CD4^+^ T cell count/μL**	666 (558−806)	685 (536−864)	0.883
**CD4^+^/CD8^+^ ratio**	1.06 (0.75−1.32)	1.03 (0.76−1.26)	0.972
**Months on treatment**	82 (75−91)	88 (77−94)	0.199
**Months in 3DR**	82 (75−91)	30 (20−38)	**−**
**Months in 2DR**	**−**	57 (53−60)	**−**

ART, antiretroiral treatment. 3DR, three-drug regimen. 2DR, two-drug regimen. MSM, men who have sex with men. DTG, dolutegravir. ABV, abacavir. 3TC, lamivudine. TDF, tenofovir disoproxil fumarate. TAF, tenofovir alafenamine. EVGc, elvitegravir/cobicistat. BIC, bictegravir. DRVc, darunavir/cobicistat. FTC, emtricitabine.

Plasma viral loads were undetectable at week 24 or earlier after initiation of treatment, most of them at week 12 (62.0%). After achieving an undetectable viral load, 1095 plasma viral loads were quantified throughout the period on 3DR [median per participant, 14 (12-18)], of which 64 (5.8%) were blips. After switching to 2DR, 526 plasma viral loads were determined [median per participant, 9 (9–12)], of which 17 (3.2%) were blips (p= 0.023).

### 3.1 CD4^+^ and CD8^+^ T-cells activation and exhaustion

**Figure 1:**
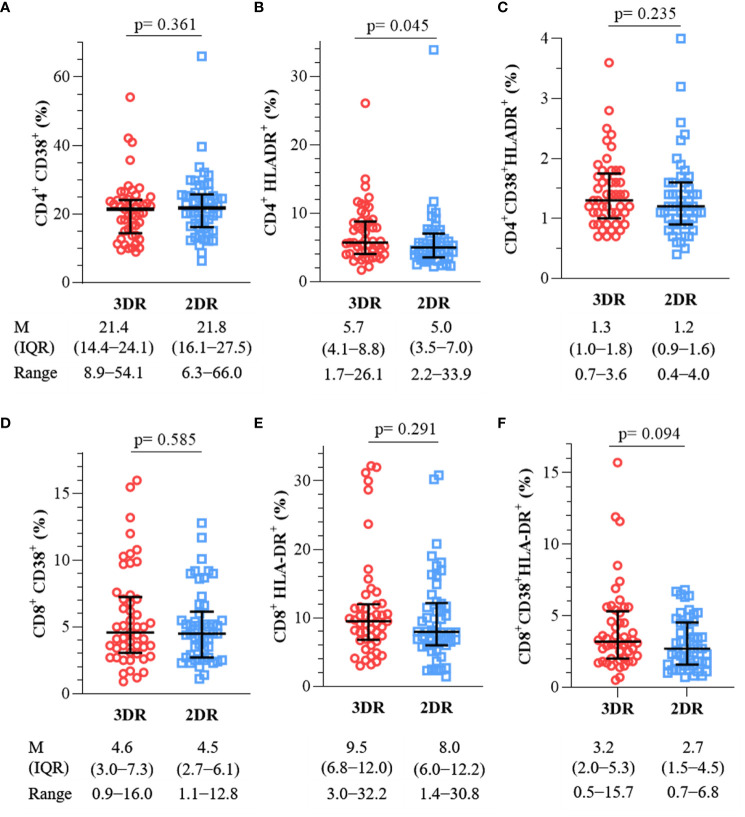
Expression of activation markers in CD4^+^ [**(A)**, CD38^+^; **(B)**, HLA-DR^+^; **(C)**, CD38^+^HLA-DR^+^] and CD8^+^ T-cells [**(D)**, CD38^+^; **(E)**, HLA-DR^+^; **(F)**, CD38^+^HLA-DR^+^]. 3DR, three-drug regimen. 2DR, two-drug regimen. M, median. IQR, interquartile range.

First, we sought differences in the expression of the activation markers CD38 and HLA-DR in CD4^+^ and CD8^+^ T cells between the 3DR and 2DR, whose values are represented in **Figures 1A–F**.

No significant differences were observed between the analyzed parameters, except for higher HLA-DR expression in CD4^+^ T cells in the 3DR; however, given its values, despite being statistically significant, it is probably of no clinical significance.

**Figure 2:**
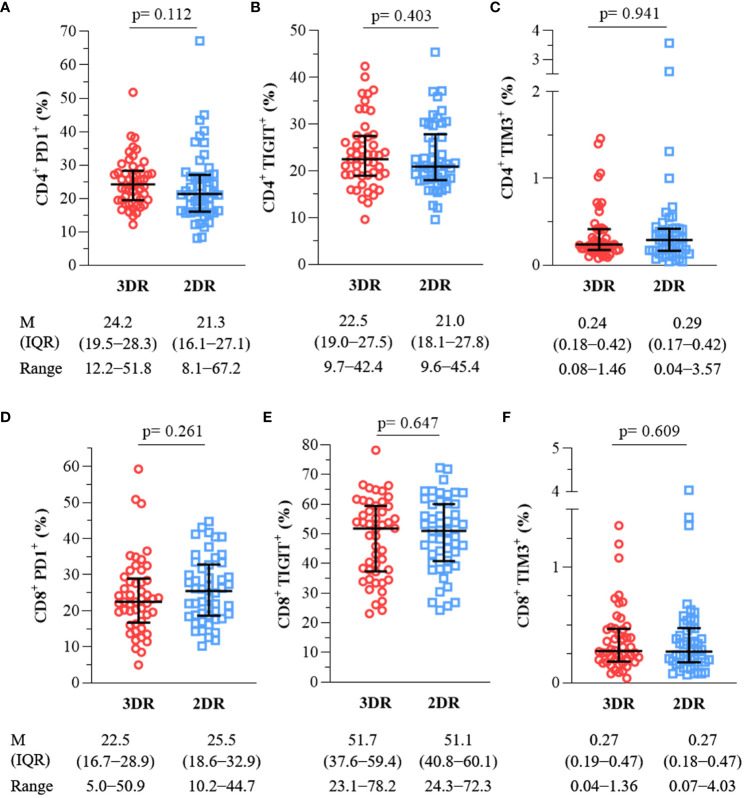
Expression of exhaustion markers in CD4^+^ [ **(A)**, PD1^+^; **(B)**, TIGIT^+^; **(C)**, TIM-3^+^] and CD8^+^ T-cells [ **(D)**, PD1^+^; **(E)**, TIGIT^+^; **(F)**, TIM-3^+^]. 3DR, three-drug regimen. 2DR, two-drug regimen. M, median. IQR, interquartile range.

On the other hand, PHIV frequently exhibits an increased expression of inhibitory receptors such as PD-1, TIGIT, and TIM-3, which contribute to progressive T-cell exhaustion. As for the previous variables, the expression of these exhaustion markers in CD4^+^ and CD8^+^ T cells did not differ between the two groups, as shown in **Figure 2**.

### 3.2 Monocyte activation and soluble inflammatory markers

Monocyte activation was measured using the soluble markers sCD14 and sCD163, which have been associated with the occurrence of SNAEs in PHIV, and MCP-1. **Table 2** shows that the three marker plasma levels were similar regardless of the treatment arm. We also measured the plasma levels of β-2M as a marker of immune-system activation and HIV infection progression.

**Table 2:** Plasma levels of soluble inflammatory markers, coagulation, and intestinal damage.

	3DR (n= 49)	2DR (n= 50)	p
**hsCRP**, mg/L	0.92 (0.45–2.23)	1.23 (0.61–2.38)	0.399
**IL-1**β, pg/mL	18.3 (11.4–25.8)	19.9 (12.8–29.6)	0.460
**IL-6**, pg/mL	2.8 (1.3–5.3)	3.2 (2.1–4.7)	0.413
**TNF-**α, pg/mL	38.2 (23.5–52.9)	42.4 (28.4–65.4)	0.214
**IFN-**γ, pg/mL	5.6 (2.5–11.1)	7.9 (3.8–12.5)	0.222
**IP-10,** pg/mL	324.4 (183.5–473.8)	296.6 (205.4–418.6)	0.790
**MIP-1**α, pg/mL	40.4 (25.2–54.3)	46.6 (26.5–65.9)	0.475
**MIP-1**β, pg/mL	37.8 (28.3–43.3)	36.6 (27.6–46.8)	0.891
**sTNFR-I,** pg/mL	284.3 (174.8–716.6)	243.0 (161.7–726.2)	0.357
**sTNFR-II,** pg/mL	2068.8 (1474.5–5650.6)	2075.7 (1425.3–5254.7)	0.493
**D-dimer,** µg/L	190.0 (150.0–370.0)	190.0 (150.0–397.5)	0.983
**P-selectin,** ng/mL	92.3 (74.4–111.6)	94.2 (79.0–110.0)	0.798
**sCD14,** ng/mL	4.5 (3.3–6.2)	5.0 (3.6–6.1)	0.531
**sCD163,** ng/mL	58.0 (38.0–84.0)	55.0 (40.1–70.3)	0.602
**MCP-1,** pg/mL	392.0 (286.6–470.3)	403.0 (306.1–552.7)	0.188
**β-2M,** mg/L	1.72 (1.49–1.95)	1.63 (1.37–1.99)	0.823
**I-FABP,** pg/mL	2146.0 (1618.0–2568.0)	2217.0 (1468.5–2700.3)	0.916
**LBP,** µg/mL	16.6 (12.9–21.5)	18.9 (15.1–22.4)	0.149

3DR, three-drug regimen. 2DR, two-drug regimen. hsCRP, high sensitivity C-reactive protein. IL-1β, interleukin-1β, IL-6, interleukin-6. TNF-α, tumor necrosis factor-α. IFN-γ, interferon-γ. IP-10, interferon gamma-induced protein 10. MIP-1α/-1β, macrophage inflammatory protein-1α/-1β. sTNFR-I/II, soluble tumor necrosis factor receptor I/II. MCP-1, monocyte chemoattractant protein 1. β-2M, β-2 microglobulin. I-FABP, intestinal fatty acid binding protein. LBP, lipopolysaccharide-binding protein.

PHIV experiences an increased incidence of SNAEs and all-cause mortality associated with cIA/I. Among the markers most related to these events are C-reactive protein, sCD14, IL-6, and D-dimer (14, 15). In addition, we measured the plasma levels of P-selectin, and the cytokines/chemokines IL-1β, TNF-α, IFN-γ, IP-10, sTNFR-I/II, and MIP-1α/1β. **Table 2** shows the detailed data for each treatment arm. Overall, the levels of these plasma biomarkers showed no differences between the 3DR and the 2DR, suggesting that cIA/I is independent of the treatment administered as long as a suppressed viremia is maintained.

Another factor linked to cIA/I is microbial translocation (16). Different markers are used to quantify gut permeability. Here, we evaluated intestinal barrier disruption by measuring I-FABP and LBP without finding differences between the two treatment groups (**Table 2**).

### 3.3 HIV-1 reservoir and transcriptional activity

**Figure 3:**
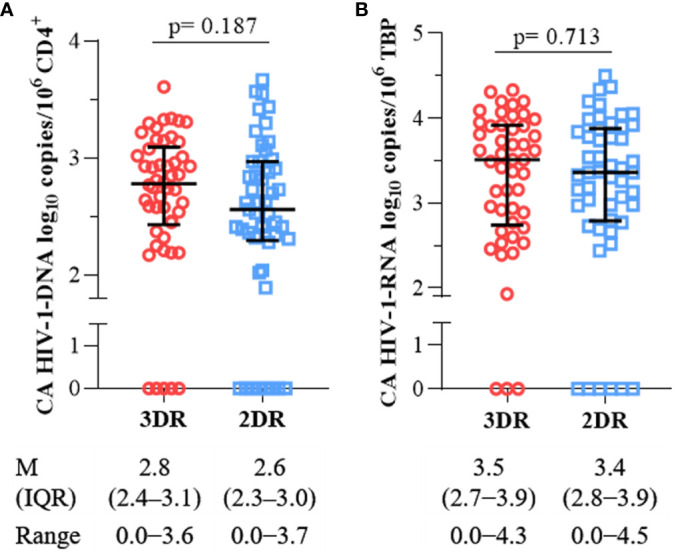
Comparison of **(A)** cellular-associated HIV-1-DNA, and **(B)** cellular-associated HIV-1-RNA between participants on a three-drug regimen (3DR) and two-drug regimen (2DR). M, median; IQR, interquartile range.

Participants who maintained 3DR exhibited a median of HIV-1-DNA of 2.8 log_10_ copies/10^6^ CD4^+^ cells (2.4–3.1) compared to 2.6 log_10_ copies/10^6^ CD4^+^ cells (2.3–3.0) in those who switched to 2DR (p= 0.187), (**Figure 3A**). As might be expected in subjects on long-term treatment with InSTI, we have not found 2-LTR circles in any participant nor those on DRVc plus 3TC.

On the other hand, current ART does not target integrated HIV-1-DNA nor prevent HIV-1-RNA transcription from the integrated provirus. However, it is possible that transcription of HIV-1-RNA and expression of antigens could stimulate the immune system and contribute to cIA/I (17). After reverse transcription of the HIV-1-RNA, the values found in both the 3DR and the 2DR were 3.5 log_10_ copies/10^6^ TBP (2.7−3.9) and 3.4 (2.8−3.9)], p= 0.713 (**Figure 3B**). Furthermore, HIV-1-DNA and RNA levels in CD4^+^ T cells were not associated with the plasma concentrations of any inflammatory marker.

Immune recovery is the ultimate goal of maintaining an undetectable viral load over time by ART.

**Figure 4:**
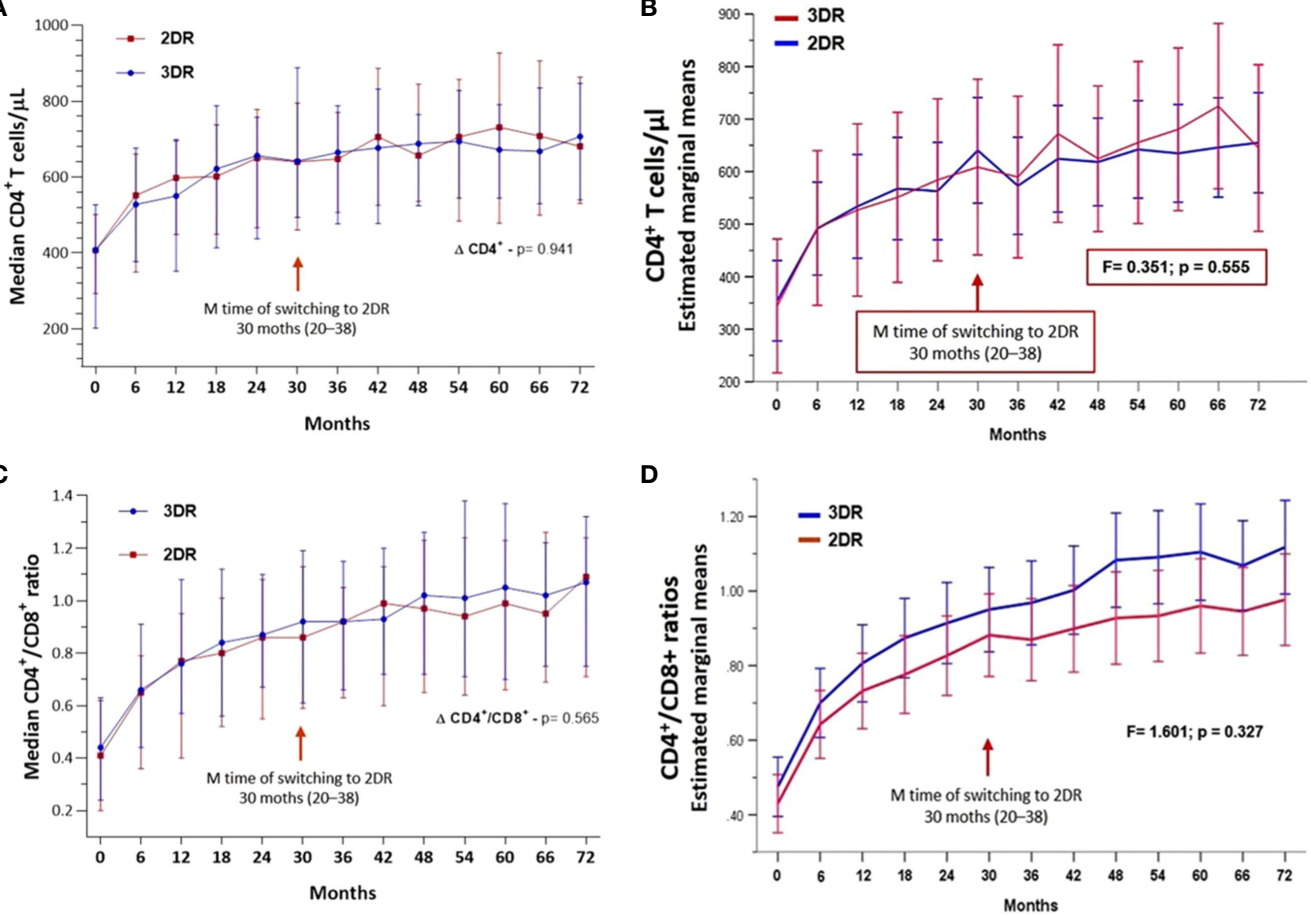
**(A)** Evolution of median CD4^+^ T cells/µL and CI_95_ in the two groups. **(B)** Evolution of median CD4^+^/CD8^+^ ratios and CI_95_ in the two groups. **(C)** Estimated marginal means of CD4^+^ T cells and CI_95_ throughout the follow-up adjusted for age, gender, risk for HIV infection, highest viral load before starting antiretroviral treatment, and number of blips during the follow-up. **(D)** Estimated marginal means of CD4^+^/CD8^+^ ratios and CI_95_ throughout the follow-up adjusted for age, gender, risk for HIV infection, highest viral load before starting antiretroviral treatment, and number of blips during the follow-up. 3DR, three-drug regimen; 2DR, two-drug regimen.

**Figures 4A, C** display the medians and IQR of CD4^+^ T-cell count and CD4^+^/CD8^+^ ratio trajectories throughout the follow-up for the 3DR and 2DR (p= 0.941 and p= 0.565, respectively), without differences between the 3DR and 2DR adjusted for possible confounders, including age, sex, risk for HIV infection, highest viral load before starting ART, and number of blips throughout the follow-up (F= 0.351, p= 0.555 and F= 1.601, p= 0.327, respectively) (**Figures 4B, D**).

## 4 Discussion

We have not observed long-term differences between maintaining a 3DR or switching to a 2DR despite a comprehensive analysis of immunological parameters, different inflammatory pathways, intestinal damage, HIV-1 reservoir, and immunological recovery.

We planned this study given the potential clinical implications of the data, first reported by Serrano-Villar et al. (18) and later published in 2022 (7, 8) endorsed by other expert opinions (5, 6), suggesting that switching to 2DR was associated with a worse long-term inflammatory profile.

Moreover, some studies have evaluated partial aspects of this topic, albeit in a shorter period than what we have done. The SALSA and TANGO trials compared CRP, IL-6, sCD14, sCD163, and D-dimer after switching to DTG/3TC versus continuing a 3DR after 48 and 148 weeks of follow-up, respectively, without differences between treatment arms (19, 20). Lombardi et al. also compared changes in CRP, IL-6, D-dimer, I-FABP, and CA HIV-1-DNA 48 weeks after switching to DTG/3TC versus maintaining 3DR without finding differences (21, 22). An additional study also evaluated changes in CA HIV-1-DNA after 48 and 96 weeks, respectively, with similar results for 2DR and 3DR based on InSTI (23).

Other clinical trials have also analyzed inflammatory biomarkers with different 2DR. The ATLAS-M compared the plasma levels of CRP, IL-6, and sCD14 at 48 weeks after switching to atazanavir/ritonavir plus 3TC versus maintaining atazanavir/ritonavir plus two NRTI (24) and the SWORD-1 and -2 trials evaluated CRP, sCD14, sCD163, IL-6, I-FABP, and acid-soluble vascular cell adhesion molecule-1 levels, at 48 weeks and up to 148 weeks after switching to DTG plus rilpivirine compared with continuing a 3DR, with no differences between treatment arms (25).

Our group has also reported comprehensive studies on this matter without finding differences in activation, proliferation, exhaustion, senescence, and apoptosis in CD4^+^ and CD8^+^ T cells, monocyte activation, plasma levels inflammatory markers, and HIV-1-specific T-cell response or the exhaustion phenotype 96 weeks after simplifying to 2DR (DTG or DRVc plus 3TC) compared with continuing an InSTI-based 3DR (26, 27). According to Llibre et al., if viremia remains suppressed, other factors could influence cIA/I in PHIV regardless of ART (25).

There are some limitations to our analysis, mainly to the inherent design of the study, which did not include an evaluation of other factors that can affect inflammation, such as comorbidities, habits, and lifestyles; however, the TRIDUAL was not designed to evaluate these aspects. Likewise, the study population mainly included male and Caucasian participants, similar to the patients seen in most centers. However, our study is the first to perform a comprehensive long-term analysis of the potential effects of the 2DR simplification compared to maintaining 3DR.

Moreover, no samples were available either from the time ART was initiated or from the time of switching to 2DR to assess the change in measurements from baseline. However, given that the characteristics of the study participants before starting ART were similar, it is plausible that there were no differences in the analyzed variables.

## 5 Conclusion

With the available evidence, including our results, it is reasonable to argue that 2DR does not negatively affect inflammatory markers or clinical outcomes compared to 3DR, neither in the medium nor in the long term, as long as undetectable viremia is maintained.

## Data availability statement

The raw data supporting the conclusions of this article will be made available by the authors, without undue reservation.

## Ethics statement

The study was approved by the ethics committee for Clinical Research of the Virgen Macarena and Virgen del Rocío University Hospitals (CEI VM-VR_06/2021_N) and the Agencia Española del Medicamento y Productos Sanitarios. The studies were conducted in accordance with the local legislation and institutional requirements. The participants provided their written informed consent to participate in this study.

## Author contributions

AS-A: Writing – original draft, Writing – review & editing. AS-G: Writing – review & editing. MT-R: Writing – review & editing. EM-M: Writing – review & editing. AA-R: Writing – review & editing. CL: Writing – review & editing. SL-F: Writing – review & editing. NE: Writing – review & editing. CR-O: Writing – review & editing. MH: Writing – review & editing. CS: Writing – review & editing. AG-V: Writing – review & editing. LL-C: Writing – original draft, Writing – review & editing.
