# Supplementary material for: Long-term effects on immunological, inflammatory markers, and HIV-1 reservoir after switching to a two-drug versus maintaining a three-drug regimen based on integrase inhibitors
Source: Front Immunol. 2024 Jul 11;15:1423734. doi: 10.3389/fimmu.2024.1423734 (PMC11269217; doi:10.3389/fimmu.2024.1423734)
Supplement: Supplementary file 1 [file DataSheet_1.docx]

**Long-term** **effects on immunological, inflammatory markers, and HIV-1 reservoir after switching to a two-drug versus maintaining a three-drug regimen based on integrase inhibitors.**

Abraham Saborido-Alconchel, MS¹, Ana Serna-Gallego, PhD¹, María Trujillo-Rodriguez, PhD¹, Esperanza Muñoz-Muela, MS¹, Ana Álvarez-Ríos, MD^2^, Carmen Lozano, PhD^3^, Silvia Llaves-Flores, BS¹, Nuria Espinosa, PhD¹, Cristina Roca-Oporto, MD¹, Marta Herrero, MD¹, Cesar Sotomayor, MD¹, Alicia Gutierrez-Valencia, PhD,^1,4^ and Luis F. Lopez-Cortes, PhD¹.

**Affiliations**

^1^ Clinical Unit of Infectious Diseases, Microbiology and Parasitology. Institute of Biomedicine of Seville/Virgen del Rocio University Hospital/CSIC/University of Seville. Spain.

^2^ Department of Clinical Biochemistry, Virgen del Rocío University Hospital, Seville, Spain.

^3^ Clinical Unit of Infectious Diseases, Clinical Microbiology and Parasitology. Virgen del Rocio University Hospital. Seville. Spain.

^4^ Primary Care Pharmacist Service, Sevilla Primary Care District, Sevilla, Spain.

**EXTENDED METHODS**

*CD4^+^ and CD8^+^ T-cell immune activation and exhaustion*

CD4^+^ and CD8^+^ T-cell activation and exhaustion were assessed by the expression of CD38 and HLA-DR, and of PD1, TIGIT and TIM-3. For this purpose, peripheral blood mononuclear cells (PBMC) were isolated using BD Vacutainer® CPT™ and stored with fetal bovine serum (FBS) and 10% dimethyl sulfoxide (DMSO) in liquid nitrogen. The day of the assay, the PBMCs were thawed, washed with PBS, and stained with different monoclonal antibodies CD3-BV650, CD4-APC/Cy7, CD8-PerCP-Cy5.5, PD1-PE, CD38-BV605, HLA-DR-FITC (BD Biosciences, USA), TIM-3-PeCy7, and TIGIT-BV421 (Biolegend, CA. USA). Viable cells were identified using Aqua (BV510) (Invitrogen). Samples were acquired on a Fortessa LSR II instrument (BD Biosciences, Madrid, Spain) and analyzed using FlowJo 9.3.2 software.

*Soluble* *inflammatory markers and intestinal damage assessment*

For plasma soluble inflammatory makers, plasma samples were aliquoted and stored at -80ºC until subsequent assays. β-2 microglobulin (β-2M) and high sensitivity C-reactive protein (hs-CRP) were quantified by an immunoturbidimetric method (Cobas 701; Roche Diagnostics). An automated latex-enhanced immunoassay (HemosIL D-Dimer HS 500, instrumentation Laboratory) was used for D-dimer. Interleukin (IL) -1β, IL-6, tumor necrosis factor-α (TNF-α), interferon-γ (IFN-γ), interferon gamma-induced protein 10 (IP-10), macrophage inflammatory protein (MIP)-1α/-1β, soluble tumor necrosis factor receptor (sTNFR) -I/-II, monocyte chemoattractant protein 1 (MCP-1), and soluble CD14 (sCD14) were measured using a multiplex bead-based immunoassay (MILLIPLEX®; Merck EMD Millipore, Billerica, MA) following the manufacturer's instructions. The assay was performed on the Luminex® MAGPIX® System detection instrument operated with xPONENT Software V4.2 (both from Luminex Corp., Austin, TX). The results were analyzed using the BelysaTM Analysis software V1.2.0 (Merck KGaA, Darmstadt, Germany). Soluble CD163 (sCD163), P-selectin, lipopolysaccharide-binding protein (LBP), and intestinal fatty-acid-binding proteins (I-FABP) were measured by immunoassay kits, following the manufacturers' instructions [Human CD163 ELISA Kit; (Thermo Fisher Scientific, Waltham, MA], Human sP-selectin ELISA kit (Thermo Fisher Scientific, Waltham, MA), Human LBP ELISA Kit (Thermo Fisher Scientific, Waltham, MA) and Human FABP2/I-FABP Quantikine ELISA Kit (R&D Systems Bio-Techne), respectively). All samples were analyzed in triplicate and repeated when the coefficient of variation (CV) was higher than 30%.


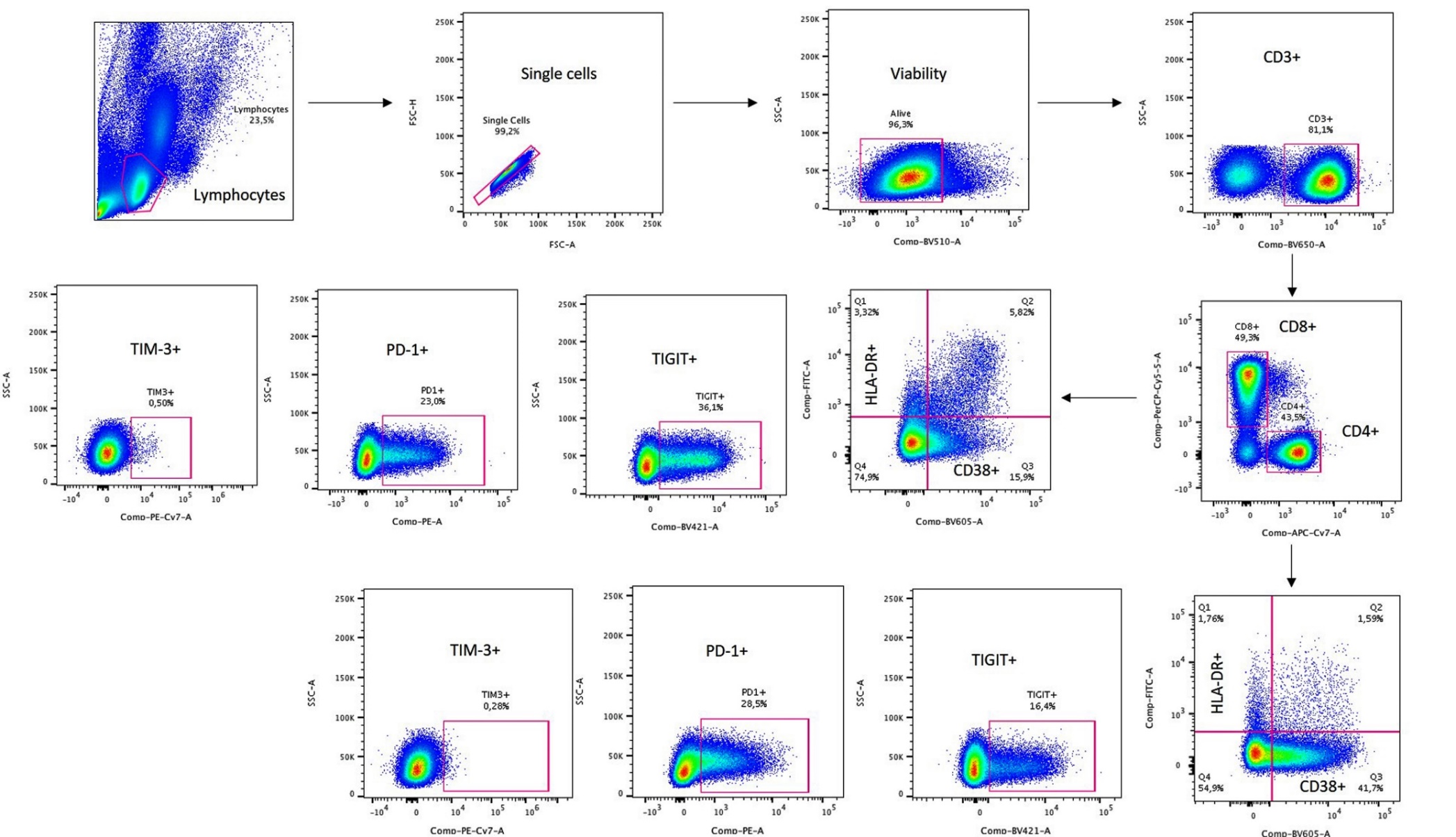
**Supplementary Figure 1.** **T-cell phenotype gating strategy.** Living lymphocytes were gated using the FSC-A/SSC-A axis, followed by a viability marker. Then, CD8+ (CD3+ CD8+) and CD4+ (CD3+ CD4+) T cells were selected. Activation (HLA-DR, CD38) and exhaustion (PD1, TIM-3 and TIGIT) markers were analyzed for each cell subset.


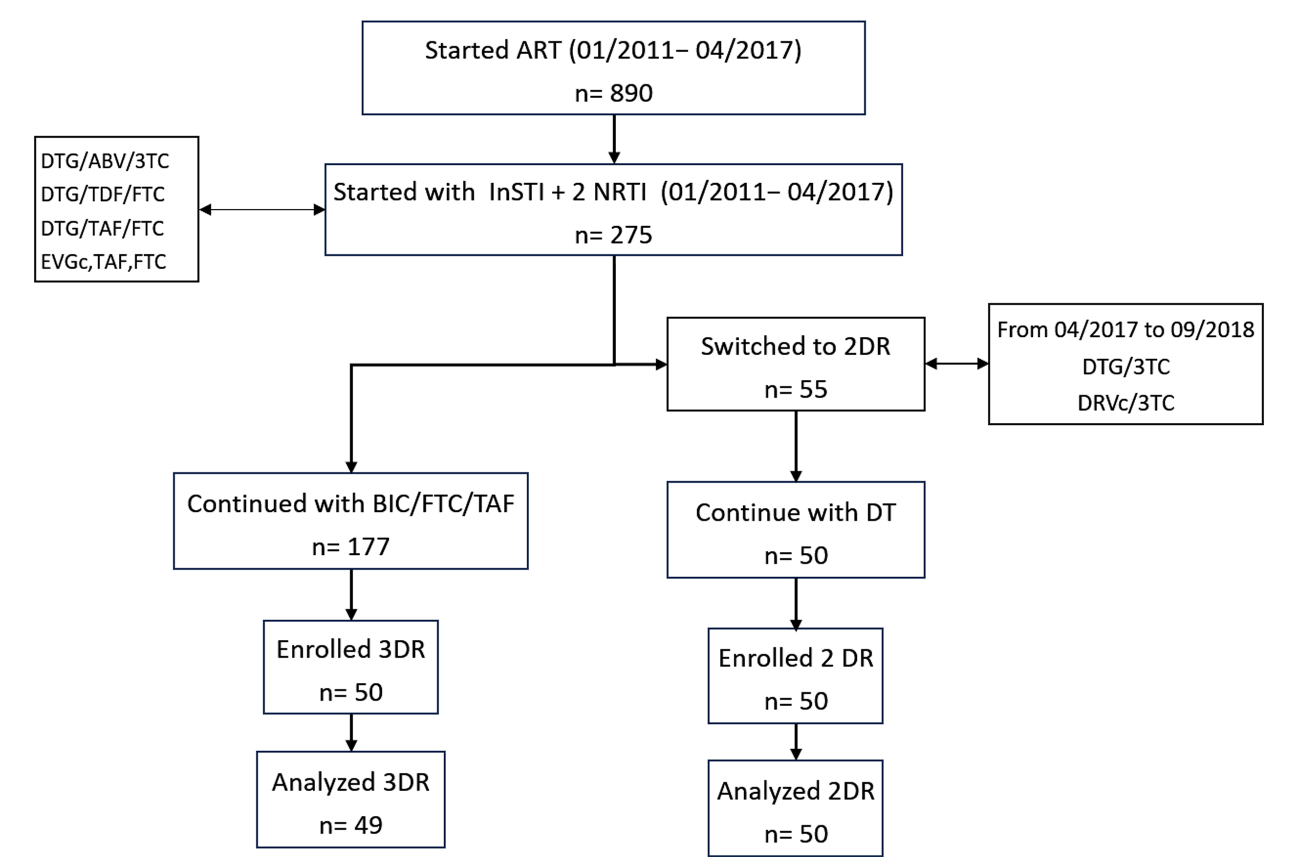


**Supplementary Figure 2.** Study profile. ART, antiretroviral treatment. InSTI, integrase strand transfer inhibitor. NRTI, nucleos(t)ide reverse transcriptase inhibitors. DTG, dolutegravir. ABV, abacavir. 3TC, lamivudine. TDF, Tenofovir disoproxil fumarate. FTC, emtricitabine. TAF, Tenofovir alafenamide. DRVc, darunavir/cobicistat. BIC, bictegravir.
